# Overview of ultrasound usage trends in orthopedic and sports physiotherapy

**DOI:** 10.1186/2036-7902-4-11

**Published:** 2012-05-28

**Authors:** Wouber Herickson de Brito Vieira, Kardec Alecxandro Aguiar, Kimberly Moreira da Silva, Pablo Miranda Canela, Flávio Santos da Silva, Bento João Abreu

**Affiliations:** 1 Departamento de Fisioterapia, Universidade Federal do Rio Grande do Norte, Av. Senador Salgado Filho, 3000 Lagoa Nova, Natal, Rio Grande do Norte, 1524-59072-970, Brazil; 2 Centro Universitário do Rio Grande do Norte, Graduação em Fisioterapia, Av. Hermes Fonseca, 789, Natal, Rio Grande do Norte, 59020-000, Brazil; 3 Departamento de Morfologia, Universidade Federal do Rio Grande do Norte, Av. Lagoa Nova S/N, Natal, Rio Grande do Norte, 1524-59072-970, Brazil; 4 Departamento de Morfologia, Centro de Biociências, UFRN, BR 101-Lagoa Nova, Natal, Rio Grande do Norte, 59072-970, Brazil

**Keywords:** Ultrasound therapy, Electrophysical agent, Survey, Physical therapy, Orthopedic

## Abstract

**Background:**

The purpose of this study is to examine current beliefs about the use, the clinical importance, the theoretical fundamentals and the utilization criteria of therapeutic ultrasound (TUS) among physical therapists on the clinical practice in orthopedic and sports physiotherapy in Brazil.

**Methods:**

A brief survey was developed based on previous studies and was sent to 55 physical therapists with advanced competency in orthopedics and sports physiotherapy. The questions addressed general topics about the professional profile and ultrasound usage and dosage.

**Results:**

Our data show the wide availability and frequent use of TUS in this sample of physical therapists. TUS is used in distinct musculoskeletal injuries and/or disorders in both acute and chronic conditions. Muscles, tendons and ligaments represented the major structures where TUS is used. Questions on the basic theory of TUS demonstrated a lack of knowledge of the ultrasound physiological effects as well as its interaction with biological tissues and TUS absolute contraindication.

**Conclusion:**

A Brazilian profile about the US usage and dosage in orthopedic and sports physiotherapy is presented and highlights the need for a continuous upgrading process and further research into its effects.

## Background

Therapeutic ultrasound (TUS) is a popular electrophysical resource that generates mechanical energy which propagates through biological tissues [1,2]. In the physical therapist practice, TUS is used to treat soft tissue injuries, accelerate the wound's repair, augment fracture healing, on dwellings resolution and to solve some bone and circulatory injuries [3-5].

Historically, TUS was used for the first time in the end of the 1940s and in the beginning of the 1950s [6-8]. During the following years, it became a therapeutic modality widely used by worldwide physical therapists [9]. Nevertheless, its great popularity over five decades, its application on clinical environment changed during this period. For example, in the past, its thermal effects were aimed primarily. Nowadays, it prioritized the non-thermal effects, especially on tissue repair and in wound healing [2]. Despite its widespread use, there is still lack of evidence of its efficacy by RCTs as well as a lack of consensus about the parameters to be used during application in treating various musculoskeletal injuries [6,10-12].

Some studies attempted to shed light into several questions regarding the use of TUS by physical therapists in their respective countries [9,12-15]. In Australia, for example, a recent study demonstrated that TUS is used daily by 84.0% of the professionals and on 25.0% of their patients [13]. In England, a survey made by applications to physical therapists of National Health Service (HNS) and private clinics showed that the TUS was used in 20.0% and 54.0% of the total interventions in the HNS and private clinics, respectively [14]. In Brazil, as well as other countries in development, there is no knowledge regarding how TUS has been used on orthopedic and sports physiotherapy (OSP) and what criteria are adopted by physical therapists during the TUS application on injuries that they need to treat daily.

Therefore, the purpose of this study was to investigate and explore current beliefs about the use, the clinical importance, the theoretical fundamentals and the utilization criteria of TUS among physical therapists in Brazil, based on a representative sample.

## Methods

### Subjects and description of the study

This research has been approved by the local committee of ethics from the University Hospital Onofre Lopes (protocol 185/2008), and it is in accordance with the direction lines within the resolution 196/96 of the Brazilian Health National Council. Previously, a brief pilot survey questionnaire was sent to three selected physical therapists to ensure it was complete, clear and objective, and if it was not, the text was adjusted accordingly. After, a survey questionnaire was designed based on the pilot survey and based on instruments applied by other authors who also attempted to examine the usage and dosage of the TUS in orthopedic, traumatologic [12] and sports physiotherapy practice [13].

The application had a total of 19 questions (objective and discursive), divided in three sections: (1) general questions, (2) specifics and (3) basic theory. Section one: questions included aspects regarding graduation and experience in OSP as well as questions about the use of TUS such as the frequency of use, importance and perceived clinical results on the following injuries: soft tissues inflammation, acute and chronic pain, reduction in tissue extensibility, delay in tissue repair, edema and difficulty of tissue remodeling. In section two, we broached questions about the biological tissues, areas of body where TUS was applied and variations in parameters (frequency, mode, intensity and time) on the injuries mentioned above. Finally, section three consisted of theoretical questions involving the TUS energy type, its interaction with biological tissues and the result of physiological effects as well as the criteria adopted for setting each one of TUS variable parameters.

Four interviewers have been properly trained and became familiar with the survey questionnaires. The interviewers visited 55 physical therapists from private clinics and from public physiotherapeutic sectors located in Brazil, which offered treatment in OSP. The minimum requirement to be considered for the study was at least of 1 year of clinical experience in the referred area. Fifty physical therapists volunteered to participate in the study and signed an informed consent. Three subjects did not return the questionnaire (*n* = 3) and are excluded from the study. Also, two physical therapists (*n* = 2) declined to participate on the study because they did not feel comfortable doing it. All physical therapists involved in this study were identified and had their names registered in a list. To obtain a probabilistic sample, 50.0% of the physical therapists working in each clinic were randomly selected to compose the sample. Those who worked in two or more clinics in common had their names removed from the list, preventing the same physical therapist from being selected more than once. In every clinic, each professional was identified by a numerical code.

After obtaining the answers, each questionnaire was delivered to an investigator to process the results and analyze the data statistically. Steps were taken to make sure that the investigator could not identify the clinic or the physiotherapist.

### Data analysis

The data obtained from the questionnaires were tabulated and the prevalence of each answer was represented by percentage values using Microsoft Excel 2003 (Microsoft, WA, USA). The correlation frequency of use of TUS and its importance, as well as the perceived clinical improvement was achieved using the Spearman's test utilizing the statistical software SPSS *for Windows*, version 11.0. To indicate statistical significance, *p* ≤ 0.05 was used.

## Results and discussion

### General questions

The physical therapists' characteristics, TUS's importance and its usage, improvement and evaluation's importance are depicted on Table 1. TUS is considered a very important resource in the clinical practice (98.0%), and most of the respondents assessed in this study use it (96.0%). Those who did not use the TUS (4.0%) justified their decision based on the fact of not knowing how to handle the resource properly and/or the use of other similar techniques. Of the physical therapists, 47.9% use TUS in at least 75.0% of their patients, and all those who use TUS reported clinical improvement (satisfactory for 64.6% and quite satisfactory for 33.3%). There is a low correlation between professional experience years and clinical improvement (*rs* = 0.2068, p value), importance and improvement (*rs* = 0.4371, p value) and frequency and importance of use (*rs* = 0.6366, p value).

**Table 1:** General characteristics of respondents, usage of TUS and perceived clinical improvement

**Variables**	**Medium (interval)**	**Number**	**Percentage**
Years of experience	4 (1–24)		
Title			
Graduate		27	54.0
Specialist		23	46.0
Master		0	0.0
Doctor		0	0.0
TUS usage			
Yes		48	96.0
No		2	4.0
Frequency of use			
Up to 10% of the patients		0	0.0
Between 10% to 25% of the patients		1	2.1
Between 25% to 50% of the patients		12	25.0
Between 50% to 75% of the patients		12	25.0
Between 75% to 90% of the patients		16	33.3
>90% of the patients		7	14.6
TUS importance			
Yes		49	98.0
No		1	2.0
Assessment of importance			
Low importance		0	0.0
Some importance		8	16.3
High importance		31	63.3
Essential		10	20.4
Improvement			
Yes		48	100.0
No		0	0.0
Improvement evaluation			
Unsatisfactory		1	2.1
Satisfactory		31	64.6
Quite satisfactory		16	33.3

**Figure 1:**
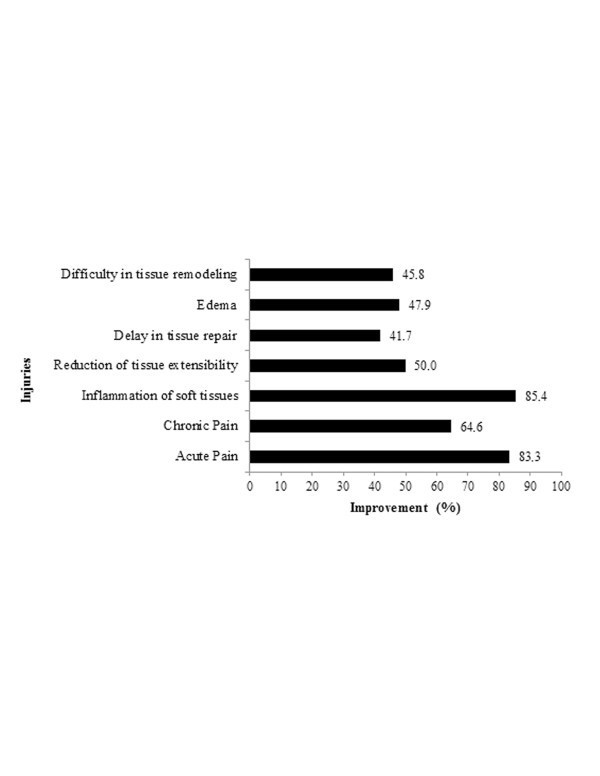
Relationship between the type of injury and clinical improvement by TUS utilization.

**Figure 2:**
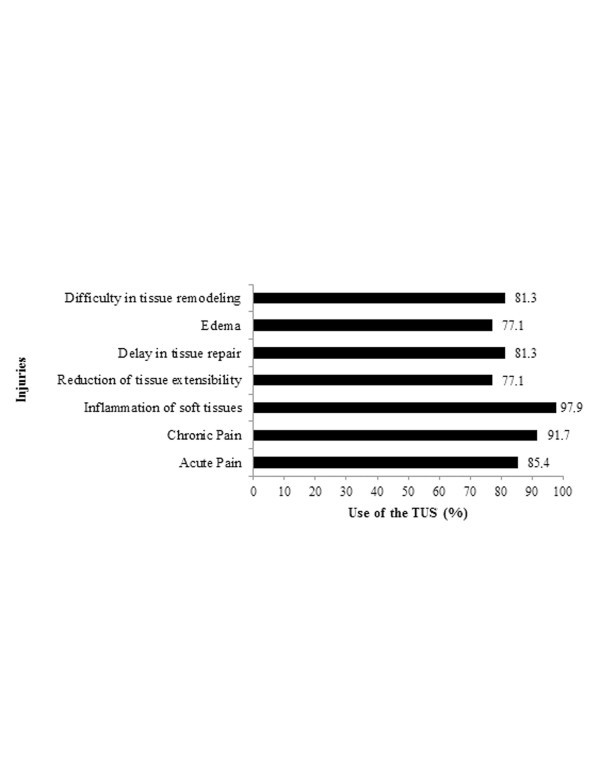
Use of TUS on different types of injuries.

The data related to the type of injury shows that TUS is widely used, and clinical improvement by application of the resource can be observed in Figures 1 and 2, respectively. TUS is widely used in distinct musculoskeletal injuries and/or disorders, in both acute and chronic conditions. In fact, 91.7% of the respondents used TUS in chronic conditions, while 85.4% utilized TUS in advance of solving acute conditions. Although questions related to clinical improvements acquired by TUS are considered empirical, they can be useful to observe the therapist's personal satisfaction about the resource. Indeed, those injuries which had the highest improvement rates corresponded to soft tissue inflammation (85.4%), acute pain (83.3%) and chronic pain (64.6%).

### Specific questions

**Figure 3:**
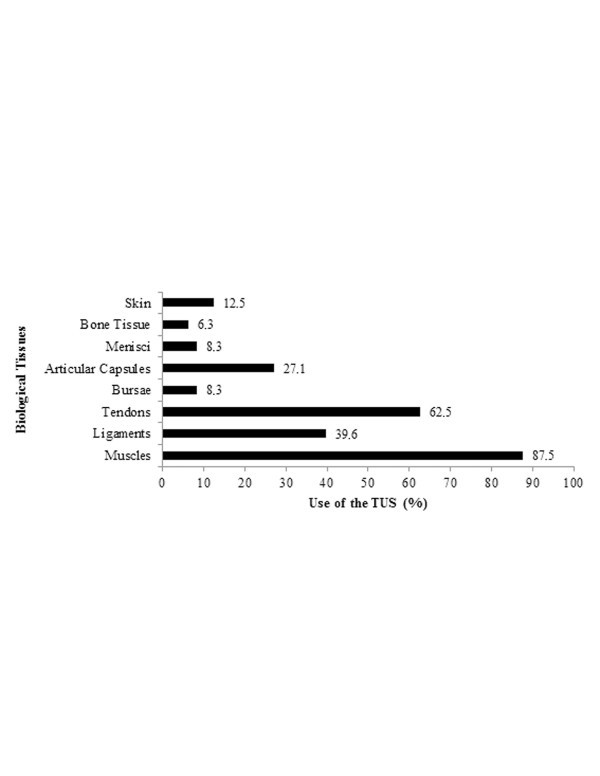
Use of TUS on different types of tissues.

**Figure 4:**
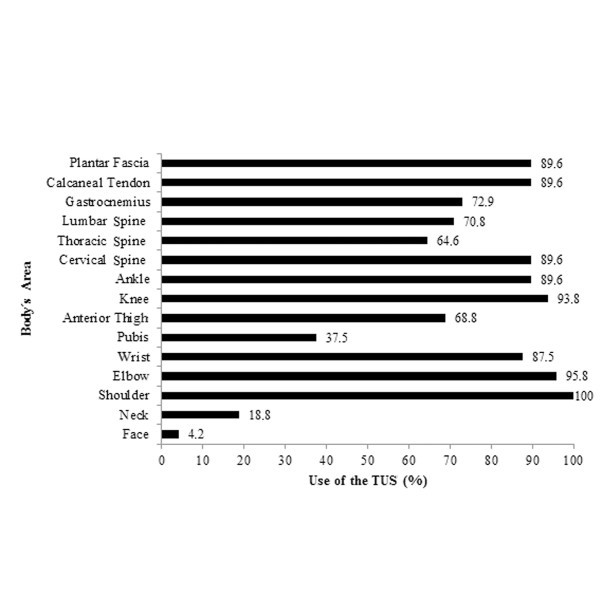
Use of TUS on different areas of the body.

In Figure 3, we can observe the tissues where TUS is most applied by the responding therapists. Bursae, menisci and bones are only treated by 8.3%, 8.3% and 6.3% of the therapists, respectively, while muscles (87.5%), tendons (62.5%) and ligaments (39.6%) represented the major structures where TUS is used. The major body regions treated by TUS are depicted in Figure 4. Shoulders represented the main body region, and its medical conditions are treated by 100% of the physical therapists who use TUS. In addition, TUS is also widely used in elbows (95.8%), knees (93.8%), plantar fascia (89.6%), calcaneus tendon (89.6%) and thoracic and cervical column (89.6%). On the other hand, the face (4.2%) and neck (18.8%) are those body regions where TUS is used least.

Table 2 displays the data about the parameters of TUS usage, which were configured for several injuries. Accordingly to the responding therapists, TUS on continuous mode is mainly used for chronic conditions, although it is still applied for acute pain in musculoskeletal tissue (12.2%) and soft tissue inflammation (28.8%). A TUS frequency of 1 MHz is the main choice for the treatment of deep tissues. However, 1 and 3 MHz are either used for superficial tissue injuries. TUS intensities varied from 0.1 to 1.0 W/cm^2^ no matter type of injury or tissue depth. Period of TUS application ranges between 2 and 4 min, and therapists prefer circular movements of the equipment's head.

**Table 2:** Parameters of TUS usage on injuries

**Variables**	**Injuries ( *N* , %)**						
	**A**	**B**	**C**	**D**	**E**	**F**	**G**
TUS modality							
Continuous	5 (12.2)	28 (63.6)	14 (29.8)	22 (59.5)	19 (48.7)	10 (27.0)	19 (48.7)
Pulse of 10 %	4 (9.8)	0 (0)	1 (2.1)	1 (2.7)	1 (2.6)	1 (2.7)	1 (2.6)
Pulse of 20 %	13 (31.7)	4 (9.1)	10 (21.3)	4 (10.8)	6 (15.4)	11 (29.7)	9 (23.1)
Pulse of 50 %	19 (46.3)	12 (27.3)	22 (46.8)	10 (27.0)	13 (33.3)	15 (40.5)	10 (25.6)
TUS frequency (superficial tissue)							
1 MHz	26 (63.4)	27 (61.4)	28 (59.6)	25 (67.6)	26 (66.7)	25 (67.6)	22 (56.4)
3 MHz	15 (36.6)	17 (38.6)	18 (38.3)	11 (29.7)	12 (30.8)	12 (32.4)	17 (43.6)
N/A	0 (0)	0 (0)	1 (2.1)	1 (2.7)	1 (2.6)	0 (0)	1 (2.6)
TUS intensity (superficial tissue)							
Between 0.1-0.5 W/cm^2^	14 (34.1)	10 (22.7)	15 (31.9)	6 (12.6)	12 (30.8)	16 (43.2)	8 (20.5)
Between 0.6-1.0 W/cm^2^	19 (46.3)	20 (45.5)	20 (42.6)	17 (45.9)	18 (46.2)	11 (29.7)	18 (46.2)
Between 1.1-1.5 W/cm^2^	4 (9.8)	7 (15.9)	7 (14.9)	9 (24.3)	4 (10.3)	4 (10.8)	3 (7.7)
Between 1.6-2.0 W/cm^2^	1 (2.4)	3 (6.8)	2 (4.3)	2 (5.4)	3 (7.7)	5 (13.5)	5 (12.8)
Between 2.1-2.6 W/cm^2^	1 (2.4)	2 (4.5)	1 (2.1)	2 (5.4)	1 (2.6)	1 (2.7)	2 (5.1)
Between 2.6-3.0 W/cm^2^	0 (0)	0 (0)	0 (0)	0 (0)	0 (0)	0 (0)	1 (2.6)
N/A	2 (4.9)	2 (4.5)	2 (4.3)	1 (2.7)	1 (2.6)	0 (0)	2 (5.1)
TUS frequency (deep tissue)							
1 MHz	38 (92.7)	38 (86.4)	43 (91.5)	32 (86.5)	33 (84.6)	32 (86.5)	31 (79.5)
3 MHz	3 (7.3)	6 (13.6)	4 (8.5)	5 (13.5)	6 (15.4)	5 (13.5)	8 (20.5)
TUS intensity (deep tissue)							
Between 0.1-0.5 W/cm^2^	5 (12.2)	1 (2.3)	4 (8.5)	1 (2.1)	6 (15.4)	4 (10.8)	5 (12.8)
Between 0.6-1.0 W/cm^2^	16 (39.0)	17 (38.6)	16 (34.0)	12 (32.4)	14 (35.9)	16 (43.2)	11 (28.2)
Between 1.1-1.5 W/cm^2^	9 (22.0)	10 (22.7)	16 (34.0)	12 (32.4)	11 (28.2)	8 (21.6)	12 (30.8)
Between 1.6-2.0 W/cm^2^	4 (9.8)	6 (13.6)	7 (14.9)	9 (24.3)	6 (15.4)	6 (16.2)	5 (12.8)
Between 2.1-2.6 W/cm^2^	4 (9.8)	7 (15.9)	4 (8.5)	2 (5.4)	2 (5.1)	3 (8.1)	6 (15.4)
Between 2.6-3.0 W/cm^2^	3 (7.3)	3 (6.8)	0 (0)	1 (2.7)	0 (0)	0 (0)	0 (0)
Time of TUS application							
Up to 2 min	1 (2.4)	1 (2.3)	2 (4.3)	2 (5.4)	2 (5.1)	1 (2.7)	2 (5.1)
Between 2–4 min	24 (58.5)	27 (61.4)	27 (57.4)	16 (43.2)	20 (51.3)	18 (48.6)	20 (51.3)
Between 4–6 min	11 (26.8)	14 (31.8)	14 (29.8)	11 (29.7)	11 (28.2)	14 (37.8)	10 (25.6)
Between 6–8 min	5 (12.2)	2 (4.5)	3 (6.4)	7 (18.9)	6 (15.4)	3 (8.1)	5 (12.8)
Between 8–10 min	0 (0)	0 (0)	1 (2.1)	1 (2.7)	0 (0)	1 (2.7)	2 (5.1)
TUS method of application							
Circular movement	38 (92.7)	41 (93.2)	46 (97.9)	33 (89.2)	35 (89.7)	35 (94.6)	34 (87.2)
Linear movement	2 (4.9)	3 (6.8)	1 (2.1)	4 (10.8)	4 (10.3)	1 (2.7)	4 (10.3)
Transducer's head turned off	1 (2.4)	0 (0)	0 (0)	0 (0)	0 (0)	1 (2.7)	1 (2.6)

A, musculoskeletal and articular acute pain; B, musculoskeletal and articular chronic pain; C, inflammation of the soft tissues; D, reduction of tissue extensibility; E, delay in tissue repair; F, edema; G, difficulty in tissue remodeling; *N*, number of subjects; %, percentage rate; N/A, no answer.

### Basic theory questions

We asked several questions regarding the basic theory of TUS usage (Table 3). Questions were asked to highlight the energy source of the TUS, its interaction with biological tissues, physiological effects and absolute contraindications of the TUS usage. The majority of therapists (24.0%) did not know the source which generates the ultrasonic energy. For the mechanisms of interaction with the ultrasonic energy with biological tissues, 42.0% answered that this interaction occurs through mechanical vibrations. Again, a high percentage of the therapists (26.0%) did not know the answer.

**Table 3:** Responses to theoretical fundamentals in TUS usage

**Variables**	**Number**	**Percentage**
Source of ultrasonic energy		
Inverse piezoelectric effect	8	16.0
Piezoelectric effect	6	12.0
Mechanical waves	3	6.0
Sound waves	7	14.0
Crystal movement	10	20.0
Interaction cell by cell	2	4.0
Electrolytic waves	1	2.0
Electric energy	1	2.0
N/A	12	24.0
Interaction of ultrasonic energy with biological tissues		
Mechanical vibration	21	42.0
Moderate discomfort	6	12.0
Sound waves	6	12.0
Through the gel	1	2.0
Chemotaxis	1	2.0
Impedance	1	2.0
Increased metabolism	1	2.0
N/A	13	26.0
Physiological effects		
Increase in cell membrane permeability	1	2.0
Increased metabolism	13	26.0
Tissue repair	14	28.0
Pro-inflammatory effect	25	50.0
Analgesia	19	38.0
Vasodilation	13	26.0
Edema reduction	5	10.0
Bone consolidation	2	4.0
Moderate discomfort	1	2.0
N/A	5	10.0
Gel importance		
Mechanical wave propagation	41	85.4
Sliding of transducer's head	2	4.2
Good functioning of the machine	1	2.1
To avoid cavitation on the transducer's head	1	2.1
To provide full contact	1	2.1
N/A	3	6.3
Absolute contraindication		
Gravidic uterus	11	22.9
Metal plates	15	31.3
Exposed wounds	10	20.8
Pacemakers	6	12.5
Gonads	5	10.4
Neoplasms	21	43.8
Epiphyseal plate	14	29.2
Carotid sinus	3	6.3
Ocular globe	2	4.2
Arthrosis	3	6.3
Osteoporosis	1	2.1
Systemic lupus erythematosus	2	4.2
N/A	2	4.2

N/A, no answer.

Curiously, questions about the physiological effects obtained by TUS presented a diverse range of answers. Some answers were conflicting, pointing pro-inflammatory effects (50.0%) as well analgesia (38.0%) and reduction of edema (10.0%). For the majority of the therapists, the gel's use facilitates the propagation of ultrasonic wave (85.4%). The majority of respondents also stated that neoplasias (43.8%) and metal plates (31.3%) are the main absolute contraindications for TUS use. These results demonstrate a lack of knowledge about the TUS's energy source, the ultrasonic waves' physiological effects, its interaction with biological tissues and the TUS's absolute contraindication.

## Conclusion

Ultrasound is considered one of the basic pillars of the electrotherapy, and although it is widely used in the clinical practice [2], there are not many data regarding the parameters used and the physiological effects acquired on specific injuries nowadays. Moreover, there is a substantial variation in ultrasound parameters selected, and while some can be supported by recent research evidence, others did not obtain positive effects in the literature [8]. In our study, we investigated the TUS usage and dosage in a sample of Brazilian therapists and explored some data evaluated previously by other researchers [12,13]. We showed that the TUS is a tool widely used by physical therapists in Brazil, and this result is in accordance with other studies that demonstrate its frequent use worldwide [9,11,12,14,16].

TUS is a therapeutic resource easy to handle and purchase, and it was present in all visited OSP clinics and offices. Also, the wide use of TUS in a number of clinical scenarios, the perception of improvement reported by therapists and patients, and the relative low cost of the treatment could justify the TUS usage by physical therapists [13]. Also, TUS is widely used in distinct musculoskeletal injuries and/or disorders, in both acute and chronic conditions. Despite the lack of evidence supporting TUS clinical efficacy, especially in humans [10,16], we found the widespread use and perceived importance of TUS among practitioners in Brazil very interesting (63.3% considered it very important, while 20.4% still considered it essential). For example, low-intensity pulsed ultrasound can be used to accelerate the fracture healing in fresh fractures and non-unions, and the evidence *in vitro* and animal studies suggests that it produces significant osteoinductive effects, accelerating the healing process and improving bone-bending strength [17]. However, the critical role of this resource for fracture healing in humans is still unknown because of the heterogeneity of results in clinical trials for fresh fractures and the lack of RCTs for delayed unions and non-unions [18]. For instance, it is reasonable to assume that the controversy about the TUS effects in bone tissues might have discouraged a large number of the respondents (93.7%) to use TUS for bone healing. In fact, this data is in accordance with the brief survey by Busse and Bandhari [19] which showed that some surgeons and physical therapy students believe that TUS is contraindicative and harmful to healing bone.

There is evidence to justify the widespread use of TUS, and one of them is related to its analgesic potential. Our findings showed that therapists noticed improvements on pain after TUS use. This result was also found by other studies that demonstrated TUS as an analgesic promoter that potentializes satisfactory outcomes for pain associated with trigger points [9,20] and decreased reported low back pain [21]. Some authors suggested that the pain improvements acquired by TUS arose from the facilitation of pro-inflammatory mediators which could enhance the whole inflammatory process [8,22,23]. Moreover, a body of evidence suggests that TUS on continuous mode is able to increase tissue temperature [24-26], and it could promote therapeutic effects from heating [26-28]. Despite these features, other therapeutic modalities could be more effective in producing heat or in reducing inflammation than the ultrasound itself [29]. For instance, laser therapy is more effective than TUS in the treatment of low back pain [21] or shoulder myofascial pain syndrome [30] in RCTs.

To avoid overlap between therapeutic tools, it is believed that the use of TUS in the clinical practice should be guided by the type of tissue injury. Ultrasound is highly effective in promoting cellular up-regulation effects in tissues that absorb more mechanical energy, those enriched of dense collagenous tissues like bones, cartilages and tendons [8]. In this work, the major regions treated with TUS were shoulders, elbows, knees and ankles and involved muscular tissue, tendons and ligaments disorders, in both acute and chronic conditions. Warden and McMeeken [13] found similar results in their study, especially for knees and ankles, although therapists reported the use of TUS on a wide range of regions as well.

The respondents reported use of TUS in both continuous and pulsate modes. The first one favors thermal effects and results in more rapid delivery of the desired energy, while non-thermal effects dominate in pulsate modes, even though these two effects occur simultaneously and can be influenced by the intensity adopted [6,14,24,27]. The frequency of 1.0 MHz was the most used regardless of the depth of the tissue. According to Watson [2], the ultrasound wave's frequency has relation with the depth reached by the ultrasonic waves in a specific biological tissue. With this in mind, the frequency of 1.0 MHz is more suitable for deep tissues, while 3.0 MHz is more appropriate for superficial tissues [31]. It is suggested that in an intermediate depth (2.5 cm), a frequency of 3.0 MHz and an intensity of 1.5 W/cm^2^ could be more effective to promote heat than 1.0 MHz [32].

On the present study, the main criteria adopted to determinate the intensity of TUS were based on the tissue depth and pathology type, while pathology type and injury phase (acute *versus* chronic) were the main criteria to adjust TUS mode. It was shown that pulsate TUS on higher intensities (0.6-1.0 W/cm^2^) was used for the treatment of soft tissue inflammation in comparison with other studies which preferred intensities between 0.3-0.5 W/cm^2^ since it focuses on TUS non-thermal effects [6,8]. Conversely, the respondents adopted the continuous mode and higher intensities (0.6-1.0 W/cm^2^ and 1.1-1.5 W/cm^2^) to treat chronic disorders, maximizing TUS thermal effects. It is suggested that continuous TUS on intensities ranging 0.8 to 1.0 W/cm^2^ is preferred for the symptoms treatment of chronic injuries and to relief pain, increase temperature and extensibility of soft tissues [33,34].

There are some aspects about the TUS usage that must be highlighted. The main standard adopted by the respondents in the present study to determine the duration of the treatment was the injury area. It is well known that the effective radiation area influences the treatment time [35], and rate of heating should be inversely proportional to injury size [29]. These theoretical aspects were rarely mentioned by the respondents. Concerning the most effective technique which should have been used to move the TUS head, clinical evidence shows that the circular movement, which was the prevalent movement pattern in the present study, is more effective since the ultrasound energy could be dissipated preferentially in a uniform way and could avoid harmful effects caused by stationary waves and cavitation [14,35]. Also, the respondents demonstrated the importance of gel use for mechanical wave propagation. In fact, it is admitted that optimal mechanical wave propagation is achieved using the gel as a coupling agent, with thinner layers offering better conductivity [36,37].

Our data showed that the TUS knowledge by the respondents is not fully satisfactory. It is difficult to point out just one or a few ‘correct’ answers when we ask about the rationale and fundaments of the TUS usage since clinical and experimental research about these issues are currently going on. However, lack of knowledge about these topics can be innocuous or potentially harmful to patients, especially when it relates to physiological effects of the TUS or its absolute contraindications [2].

In summary, the present study demonstrates the widespread use and the importance of the TUS on clinical practice of OSP in Brazil. The results found on dosage use and adopted criteria showed a relative coherence on this resource's use, although the basic theory was not fully satisfactory. A continuous process of professional updating is suggested to confirm these clinical results obtained by physical therapists as well as more evidence-based data to assess the effectiveness of ultrasound as a therapeutic resource in specific musculoskeletal conditions.

## Competing interests

The authors declare that they have no competing interests.

## Authors' contributions

WHBV participated in the study design, data acquisition, data analysis, and manuscript writing, and approved the publication of this study. KAA, KMS, and PMC participated in data acquisition, data analysis, and manuscript writing, and approved the publication of this study. FSS participated in data acquisition, study design and manuscript revision for important intellectual content, and approved the publication of this study. BJA participated in study design, data analysis and manuscript writing, and approved the publication of this study. All authors read and approved the final manuscript.
